# The Threshold of Admission Glycemia as a Predictor of Adverse Events in Diabetic and Non-Diabetic Patients with Acute Coronary Syndrome

**DOI:** 10.4137/cmc.s2289

**Published:** 2009-04-01

**Authors:** Taysir S. Garadah, Salah Kassab, Qasim M. Al-Shboul, Abdulhai Alawadi

**Affiliations:** 1 Cardiac Unit, Salmaniya Medical Complex.; 2 College of Medicine and Medical Sciences, Arabian Gulf University, Manama, Kingdom of Bahrain.

**Keywords:** stress hyperglycemia, acute myocardial infarction, glycosylated hemoglobin

## Abstract

Recent studies indicated a high prevalence of hyperglycemia in non-diabetic patients presenting with acute coronary syndrome (ACS). However, the threshold of admission glucose (AG) as a predictor of adverse events in ACS is unclear.

**Objective:**

The aim of this study was to assess the threshold of admission glucose (AG) as a predictor of adverse events including Major Acute Cardiac Events (MACE) and mortality, during the first week of admitting patients presenting with ACS.

**Material and Methods:**

The data of 551 patients with ACS were extracted and evaluated. Patients were stratified according to their blood glucose on admission into three groups: group 1: ≤7 mmol/L (n = 200, 36.3%) and group 2: >7 mmol/L and <15 mmol/L (n = 178, 32.3%) and group 3: ≥15 mmol/L (n = 173, 31.4%). Stress hyperglycemia was arbitrarily defined as AG levels > 7 mmol/L (group 2 and 3). Patients with ACS were sub-divided into two groups: patients with unstable angina (UA, n = 285) and those with ST segment elevation myocardial Infarction (STEMI, n = 266) and data were analyzed separately using multiple regression analysis.

**Results:**

The mean age of patients was 59.7 ± 14.8 years and 63% were males. The overall mortality in the population was 8.5% (5.4% in STEMI and 3.1% in UA) patients. In STEMI patients, the odds ratio of stress hyperglycemia as predictor of mortality in group 3 compared with group 1 was 3.3 (CI 0.99–10.98, *P* < *0.05*), while in group 2 compared with group 1 was 2.4 (CI: 0.75–8.07, *P* = *0.065*) after adjustment for age and sex. Similarly, in UA patients, the odds ratio of stress hyperglycemia in group 3 compared with group 1 was 2.7 (CI 0.37–18.98, *P* < *0.05*), while in group 2 compared with group 1 was 2.4 (CI: 0.4–15.2, P = 0.344) after adjustment for age and sex. The incidence of more than 2 MACE in both STEMI and UA patients was higher in group 3 compared with the other two groups. Regression analysis showed that history of DM, high level of LDL cholesterol, high level of HbA1c, and anterior infarction were significant predictors of adverse events while other risk factors such as BMI, history of hypertension and smoking were of no significance.

**Conclusion:**

This study indicates that the stress hyperglycemia on admission is a powerful predictor of increased major adverse events and hospital mortality in patients with acute coronary syndrome.

## Introduction

Admission Hyperglycemia (AG) is commonly associated with marked increase of mortality in patients hospitalized with acute coronary syndrome (ACS). Despite the fact that several studies have documented this association, hyperglycemia remains underappreciated as a risk factor, and it is frequently untreated in ACS patients. This is in large due to the limitations of prior studies regarding the definition of AG and to our understanding of the relationship between hyperglycemia and the poor outcomes.^1^–^3^

The predictive value of admission hyperglycemia in acute myocardial infarction whether the patient is diabetic or not has been shown to be conflicting in the previous reports.^4^,^5^ Recent data indicated a high prevalence of abnormal glucose metabolism in patients with no history of DM at the time of acute myocardial infarction (AMI), (stress hyperglycemia).^6^

The level of glycosylated hemoglobin (HbA1c) in patients with myocardial infarction reflects the glycemic control level within a period of time up to 120 previous days and was shown to segregate patients who had only stress hyperglycemia during acute infarction from those who are diabetic^7^. So far in the setting of ACS, the predictive role of serum level of HbA1c as an independent predictor of mortality is not clear as it was reported as a useful prognostic marker in some reports, but negative by others.^7^,^8^

The first objective of this retrospective study was to define hyperglycemia on admission and to assess the threshold of blood level where it has an adverse impact on the outcomes in patients with ACS. The second objective was to determine whether hyperglycemia on admission (AG) in non diabetic patients (stress hyperglycemia) is an independent factor for in-hospital mortality in patients with ACS or just a surrogate of great severity.

## Patients and Methods

### Study sample

The data of five hundred and fifty one (551) patients who were admitted to the coronary care unit (CCU) in Salmaniya Medical Complex in Bahrain was extracted from the patients’ files. The study had institutional approval.

### Inclusion criteria

Patients were eligible for entry in the study if they have retrosternal chest pain on rest for more than 20 minutes with ST segment elevation of more than one millimeter in two contiguous leads and two fold increment of the cardiac enzymes creatine kinase (CK) and CK iso-enzyme (CKMB) classified as ST elevation myocardial infarction, (STEMI) or with ST segment depression without increase of cardiac enzymes, (classified as Unstable angina (UA). The data of all patients admitted to the CCU that fit the inclusion criteria were extracted over the duration of 12 months (from beginning of January to end of December 2005).

### Exclusion criteria

Patients were excluded if they had history of severe heart failure or if they had history of definite myocardial infarction in the past.

### Clinical and biochemical data

Patients’ data of age, sex, body mass index, history of diabetes mellitus, hypertension, smoking and hyperlipidemia (LDL > 3.9 mmol/L) were all extracted. Serum levels of the following parameters were tabulated: Serum admission glucose (AG), glycosylated haemoglobin (HbA1c), low density lipoprotein (LDL) cholesterol and total creatine kinase as previously reported.^9^

**Figure 1.**
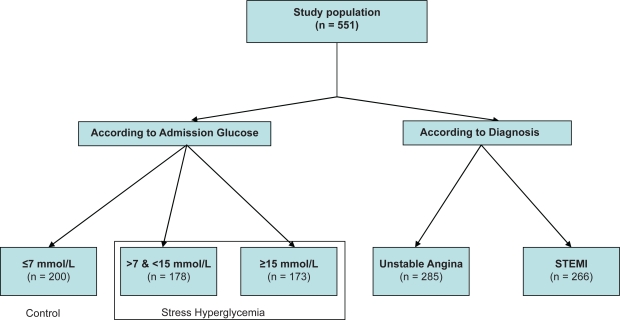
Distribution of the population groups in the study.

Patients were stratified according to their blood glucose on admission into three groups as shown in Figure 1 Group 1 (control): ≤7 mmol/L (n = 200, 36.3%) and group 2: >7 mmol/L and <15 mmol/L (n = 178, 32.3%) and group 3: ≥15 mmol/L (n = 173, 31.4%). Stress hyperglycemia was arbitrarily defined for AG levels >7 mmol/L (group 2 and 3, n = 351, 63.7%). Patients with ACS were subdivided into two groups: patients with unstable angina (UA, n = 285) and those with ST segment elevation myocardial Infarction (STEMI, n = 266).

The level of HbA1c of more than 6.5% was regarded high and indicative of DM in the past even if patient was not aware of it. Thrombolytic therapy in STEMI patients and other medications given were recorded. The frequency of using different medications during hospitalization in all groups were matched for oral aspirin, oral clopidogrel, intravenous heparin, subcutaneous low molecular weight heparin, beta blockers, angiotensin receptor antagonists, and intravenous nitroglycerine.

### Outcomes

Events such as death or Major Acute Cardiac Events (MACE) such as significant arrhythmias (supra ventricular tachycardia, atrial fibrillation, ventricular tachycardia or ventricular fibrillation), pulmonary edema, hypotension (if systolic pressure is less than 90 mmHg) or cardiogenic shock were all extracted and tabulated in groups according to plasma AG level on admission as mentioned above.

### Twelve leads ecg

The site of myocardial infarction on 12 leads ECG was analyzed. ST segment elevation at leads V1–V4 represented anteroseptal infarction, at leads II, III, aVF represented inferior infarction and at leads I, aVL, V5, V6 represented lateral infarction.

### Statistical analysis

The extracted data of the patients were entered and analyzed using the Statistical Package for Social Sciences program (SPSS version 15.0). Relevant descriptive statistics were computed for all study variables. One-way analysis of variance (*ANOVA*) was used to compare the means of the quantitative variables (LDL, Hb, creatinine, BMI and age) across the three groups based on serum level of AG. *Post hoc* analysis was used for multiple comparisons. The association between the groups of AG and each of the study qualitative variables such as: history of smoking, history of DM, history of hypertension, gender and MACE was tested using the *Chi-square* test of independence. The multiple logistic regression analysis was used to predict the probability of death using the following predictors Serum level of AG, HbA1C, LDL level, BMI, history of hypertension, DM, or smoking and the site of myocardial infarction. The odds ratios with the confidence intervals (CI) were calculated for all variables. Any analysis with *p*-value less than 0.05 was considered statistically significant.

## Results

The data of 551 patients was evaluated in the study. Fifty one percent had myocardial infarction (STEMI, n = 285), and 49% had unstable angina (UA, n = 266) with no infarction. Three hundred and fifty one patients (63.7%) were hyperglycemic with AG more than 7 mmol/L and 200 (36.3%) were normoglycemic with AG less than 7 mmol/L.

Among the hyperglycemic patients, 186 (33.8%) had DM and, 186 (33.8%) had no DM, 271(49%) had history of hypertension and 211 (38.2%) had history of smoking.

Table 1 shows the demographic and biochemical data of the study population. The mean age was 59.7 ± 14.8 years (range 24–88), with 348 males (63%). There were no significant differences between males and females in relation to the level of AG, serum creatinine, level of hemoglobin and body mass index.

The LDL level and the HbA1c level were matching in each subgroup with increment at group 2 and 3 compared to group 1 in SEMI and in UA groups. Multiple comparison results show that the mean level of total CK was significantly higher in group 3 compared to group 1, and group 3 versus group 2 (*p* < 0.001 in each).

The mean HbA1c was significantly lower in group 1 compared with that of groups 2 and 3 (*p* < 0.001 in each). The mean age of group 3 was significantly higher than that of group 2 (*p* = 0.027). The serum LDL level for the three groups were significantly different (*p* < 0.001 in each).

In STEMI group, 80/112 (72%) of patients in group 1, 60 out of 88 (69%) patients in group 2 and 59 out of 85 (70%) in group 3 were given thrombolytic therapy.

The site of AMI on 12 leads ECG in STEMI group was anterior in 50%, 52% and 54% of patients in group 1, 2, 3 respectively and was inferior in 31%, 33% and 27% of the patients in the three groups, respectively. The sites of AMI were evaluated among those who died, it was observed that the site was anterior MI in 22/30 patient (73%), inferior lateral in 4 (13%) and inferior with right ventricular infarction in 4 (13%).

### In-hospital outcomes

#### Major acute cardiac events (mace) in stemi patients

**Figure 2.**
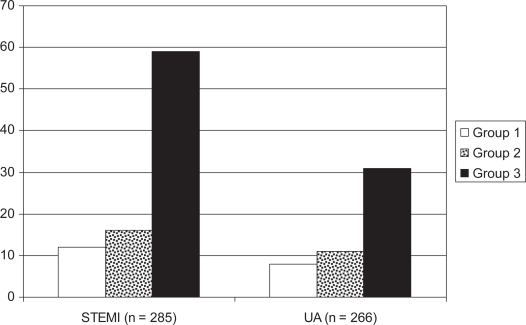
The number of patients who had more than two major acute cardiac events (MACE) in the study population. Group 1: control group with glucose ≤7 mmol/L, Group 2: admission glucose 7−≤15 mmol/L and Group 3: admission glucose >15 mmol/L.

Figure (2) shows the frequency of MACE in the three groups in STEMI and UA patients. The number of patients with STEMI who had more than two MACE were, 59 (67% and 16 (17%), 12 (8%) in groups 3, 2 and 1, respectively. In unstable angina, patients who had more than 2 MACE were 36 (36%), 11(12%) and 8 (7%) in groups 3, 2 and 1, respectively.

#### Mortality in STEMI patients

Overall mortality in the population was 47/552 (8.5%), where 30/552 (5.4%) in STEMI and 17/552 (3.1%) in UA. In the STEMI patients, the total death was 30: twelve patients (4.2%) were in group 3, eleven patients (3.9%) in group 2 and seven (2.4%) in group 1. In UA patients the total death were 17 out of 266, nine patients (3.3%) in group 3, six patients (2.2%) in group 2 and two in group 1 (0.7%), respectively.

Table 2 shows the results of multivariate regression analysis for predictors of mortality in the study population. In STEMI group, the odds ratio of serum level AG, HbA_1_C, and LDL were significant (p < 0.05), the odds ratio of the anterior MI (not shown in Table) on 12 leads ECG was 2.7 (CI: 0.5–13, *p* < 0.001), the odds ratio for inferior AMI and other sites were not significant. The stress hyperglycemia, level of LDL, serum level of HbA1c and anterior AMI on ECG were significant predictors of adverse events. The BMI, history of hypertension and smoking on admission were not significant. The odds ratio of serum level of HbA1c as a predictor of mortality in the whole study was 1.4 (CI: 0.5–1.9, *p*-value = 0.04) in the hyperglycemia patients.

Table 3 shows the predictive value of stress hyperglycemia and D.M. for mortality in groups 3 and 2 compared with group 1 after adjusting for age and gender. In stress hyperglycemia, the odds ratio of mortality in group 3 compared to group 1 was 3.3 (CI: 0.99–10.98, *p* < 0.05) in STEMI patients and 2.7 for UA (CI: 0.3–17.4, *p* < 0.05). In group 2 compared to group 1, the odds ratio were not significant for neither STEMI of 2.4 (CI: 075–8.07, *P* = 0.318) nor for UA of 2.4 (CI: 0.37–15.2, *p* = 0.34).

In hyperglycemia with history of DM, the odds ratio were significant in group 3 in STEMI in comparison to group 1, were of no significance in UA.

The odds ratio for death was noted to be progressively increased with a higher level of AG.

## Discussion

This study demonstrates that stress hyperglycemia on admission in the absence of DM is a significant predictor of adverse outcome in patients presenting with ACS. We have also demonstrated that serum level of HbA1c and anterior AMI on ECG are significant predictors of adverse outcome in patients presenting with ACS. However, BMI, level of LDL, history of hypertension and smoking on admission were not significant predictors of adverse outcome.

In the current study population, the incidence of stress hyperglycemia was 33%, which is lower than previous reports of 48% and 54%.^10^,^11^ Those who had hyperglycemia and DM were 29%. The incidence of hyperglycemia with DM varies in different studies between 23%–41%.^12^,^13^ Thirty two percent had history of hypertension, which is higher than those previously reported (28% and 25%) in other studies, respectively.^14^,^15^

The unique finding in this study is that we demonstrated the relationship between adverse events in ACS patients and admission glucose based on a unique classification of stress hyperglycemia. The stratification for the hyperglycemia was based on AG at two levels, group 2 between 7 and 15 mmol/L, and group 3 is more than 15 mmol/L, whereas AG less than 7 mmol/L was regarded as normoglycemia. In some other studies, however, clinical analysis for outcome was based on AG level below or above 9.4 mmol/L.^16^ In two other studies, the patients data were analyzed based on the normal level of <6.8 mmol/L and above 11 mmol/L, and the in-between of 6.8 to 11 mmol/L.^17^,^18^ The differences in selecting the cutoff for the stress hyperglycemia between the current study and others appear to be related to the research question raised in each study. The stratification in our study was arbitrarily selected with the aim of assessing the threshold of the AG level and its relationship with adverse outcome in ACS patients.

In ACS patients with stress hyperglycemia, the odds ratio of in-hospital mortality was significantly higher in group 3, compared with group 1 in STEMI and in UA patients. Also in group 2, the odds of mortality were higher but with no significance compared with group 1 for STEMI and UA patients. This was in keeping with many other trials that showed stress hyperglycemia as a predictor of in hospital mortality.^19^,^20^ The cardiac events of more than two per patient were higher in group 3 and group 2 compared with group 1 in both STEMI and UA patients. This is in agreement with a previous report showing that cardiac failure was significantly higher among those with high level of AG on admission.^21^,^22^ Another study have also demonstrated that the relative risk of congestive cardiac failure in AMI with stress hyperglycemia had increased risk of death with relative risk of 1.7 (CI: 1.2–2.4).^21^,^22^

In STEMI patients, the level of the cardiac enzyme creatine kinase were significantly higher in group 3 compared to group 1, indicating a larger myocardial infarction in that group. It is not known whether this was the cause or the result of high AG. The rate of anterior AMI was high among those who died (73%). In one report, the high level of total CK was associated with high level of glucose and that the site of anterior MI was an independent predictor for high in-hospital complications in AMI patients.^15^ Patients with stress hyperglycemia in this study had a similar and significant predictive odds ratio for mortality compared with patients with DM in STEMI patient and UA patients when the level of AG > 15 mmol/L. Previous reports have shown that hyperglycemia on admission with history of DM is a useful predictor of short and long-term morbidity and mortality.^24^,^25^

The exact mechanism of the high predictive value of stress hyperglycemia on admission for adverse outcome in ACS is not known. However this could be due to high adrenergic drive in ACS during myocardial ischemia.^24^ Many previous experimental and human studies showed that the ischemic preconditioning, which is a protective mechanism against myocardial injury on exposure to ischemia is blunted or abolished completely in linear relation with the level of glucose.^25^ The high level of AG has recently been linked to a greater tendency for local thrombin generation and platelet activation and unfavorably altered clot features in patients with ACS.^26^ An alternative approach in discussion is that stress hyperglycemia could be a consequence of the ACS. The cause of stress hyperglycemia in ACS could be due to increased activity of neurohormonal pathways such as catecholamines, cortisol and growth hormone which produce significant insulin resistance.^27^ The activation of circulating cytokines such as tumor necrosis factor-α may lead to reduction of insulin sensitivity, thus increase serum level of glucose.^28^,^29^ Investigating a cause and effect relationship between stress hyperglycemia and ACS requires other future studies with different design.

High level of glycosylated hemoglobin in this study was shown to be an independent predictor of adverse outcome in the setting of acute myocardial infarction which is in agreement with others^7^,^8^ but differs with Timmer et al. where HbA1c was of no predictive value for mortality or morbidity in ACS patients.^11^ It was presumed that high level of HbA1c is a leading cause of endothelial dysfunction and impairment of the mechanism for the release of ischemia mediators.^30^

Among other factors that was found to be of adverse predictive value on regression analysis was the anterior MI, and the history DM. History of hypertension on admission as a predictor of mortality in STEMI was not a significant, similar finding was reported previously.^31^ The mean of the body mass index was not elevated indicating the obesity was not noted in the study group and the odds of BMI were not significant as a predictor of poor outcome. However in one study it was shown high BMI on admission is related positively to occurrence of ACS in patients with well established coronary artery disease.^32^

## Limitations of the Study

The retrospective nature of the study and the shortterm outcome measurements could be considered as limitations in the current study.

## Conclusion

Multivariate logistic regression analysis in this study showed that the stress hyperglycemia on admission is a powerful predictor of in-hospital mortality in patients with acute ST segment elevation myocardial infarction and unstable angina, particularly when the level of AG is > 15 mmol/L. The history of DM, the site of anterior MI on ECG, high LDL cholesterol and high level HbA1c were all useful predictors of adverse outcome. However, the history of smoking, history of hypertension and Body mass index on admission were not useful predictors of adverse outcome in ACS.

## Figures and Tables

**Table 1.** Demographic and biochemical data of the study groups (n = 551) according to admission glucose.

**Parameters**	**STEMI (n = 285)**			**UA (n = 266)**		
	**Group 1 (n = 112)**	**Group 2 (n = 88)**	**Group 3 (n = 85)**	**Group 1 (n = 88)**	**Group 2 (n = 90)**	**Group 3 (n = 88)**
Mean admission glucose (mmol/L)	5.04 ± 0.89	9.62 ± 2.15	22.25 ± 10.80*	5.3 ± 0.89	9.9 ± 2.15	20.2 ± 10.80*
Age (years)	58.0 ± 16.5	55.2 ± 19.2	61.9 ± 11.7*	57.1 ± 15.4	59.1 ± 17.1	57.2 ± 11.7
Sex (Male)	73	55	54	56	55	55
BMI (Kg/m^2^)	26.47 ± 3.53	25.95 ± 3.70	26.40 ± 3.61	26 ± 3.53	26 ± 3.70	26 ± 3.61
History of smoking	37	41	33	28	40	32
History of hypertension	46	44	48	44	46	43
Serum total CK (U/L)	1126.28 ± 927	1190.72 ± 827	3447.35 ± 1729*	130.23 ± 32	178.45 ± 24	145.34 ± 19
Serum creatinine (μmol/L)	95 ± 16	99 ± 11	106 ± 26	99 ± 15	89 ± 14	110 ± 24
Serum HB (g/dL)	11.1 ± 1.1	12.0 ± 1.1	11.7 ± 1.5	10 ± 1.1	10.3 ± 1.1	9.9 ± 1.5
Serum LDL (mmol/L)	4.2 ± 1.1	4.91 ± 0.7	6.6 ± 0.8*	4.6 ± 1.1	6.8 ± 0.7	5.5 ± 0.8*
Mean of HbA1c (%)	4.2% ± 2.2%	7.1% ± 1.9%	9.9% ± 2.9%*	4.6% ± 2.2%	6.5% ± 1.9%	6.3% ± 2.9%*
History of DM prior to admission	0	39	45	0	38	43

**Abbreviations:** STEMI, ST segment elevation myocardial infarction; UA, unstable angina.

(*) P-value, 0.001.

**Table 2.** Multiple logistic regression analysis results for the prediction of death in groups with hyperglycemia against group 1 after adjusting for age, gender and history of DM.

**Group according to Admission glucose**	**STEMI group**			**UA group**		
**Variable**	**Odds ratio**	**Confidence interval**	***p*-value**	**Odds ratio**	**CI**	***p*-value**
**Admission glucose**	2.8	0.7–11.3	0.03	2.8	0.8–10.8	0.02
**HbA1c**	1.4	0.5–1.9	0.04	0.96	0.7–1.2	0.03
**History of smoking**	0.5	0.2–1.5	0.27	0.71	0.2–2.8	0.56
**History of Hypertension**	0.8	0.3–1.6	0.35	1.3	0.4–4.3	0.65
**BMI**	1.01	0.9–1.1	0.77	0.9	0.78–1.08	0.313
**LDL level**	1.01	0.7–1.1	0.03	0.99	0.4–1.4	0.04

**Abbreviations:** STEMI, ST segment elevation myocardial infarction; UA, unstable angina; HBA_C_, Glycosylated hemoglobin C; BMI, body mass index.

**Table 3.** The odds ratio of deaths in patients with stress hyperglycemia with no history of DM on admission and hyperglycemia with history of DM.

**Parameter**	**Group 2 versus 1**			**Group 3 versus 1**		
	**Odd ratio**	**C I**	***p*-value**	**Odds ratio**	**C I**	***p*-value**
**STEMI**						
with stress hyperglycemia	2.4	0.75–8.07	0.318	3.3	0.99–10.98	0.04
with history of DM	3.1	0.94–8.07	0.06	3.2	0.99–10.98	0.03
**UA**						
with stress hyperglycemia	2.4	0.4–15.2	0.34	2.7	0.37–18	0.02
With history of DM	2.7	0.4–173	0.28	4.5	0.3–25.7	0.08
